# Molecular epidemiology of *Acinetobacter baumannii* complex causing invasive infections in Korean children during 2001–2020

**DOI:** 10.1186/s12941-023-00581-3

**Published:** 2023-05-03

**Authors:** Hyun Mi Kang, Ki Wook Yun, Eun Hwa Choi

**Affiliations:** 1 Department of Pediatrics, College of Medicine, Seoul National University, Seoul, South Korea; 2 Department of Pediatrics, College of Medicine, The Catholic University of Korea, Seoul, South Korea; 3 Department of Pediatrics, Division of Pediatric Infectious Diseases, Seoul National University Children’s Hospital, Seoul, South Korea

**Keywords:** *Acinetobacter*, Children, Genotype, Resistance

## Abstract

**Background:**

*Acinetobacter baumannii* (AB) has emerged as one of the most problematic pathogens affecting critically ill patients. This study aimed to investigate the longitudinal epidemiology of AB causing invasive diseases in children.

**Methods:**

*Acinetobacter* spp. cultured from sterile body fluids and identified as *Acinetobacter calcoaceticus*-*baumannii* (ACB) complexes by automated systems from children aged below 19 years old were prospectively collected during 2001–2020. The discriminative partial sequence of *rpoB* gene was sequenced to identify the species, and sequence types (STs) were determined. Temporal changes in antimicrobial susceptibilities and STs were analyzed.

**Results:**

In total, 108 non-duplicate ACB isolates were obtained from patients with invasive infections. The median age was 1.4 (interquartile range, 0.1–7.9) years, and 60.2% (n = 65) were male. *Acinetobacter baumannii* comprised 55.6% (n = 60) of the isolates, and the 30-day mortality was higher in patients with isolated AB than in those with non-*baumannii Acinetobacter* spp. (46.7% vs. 8.3%, *P* < 0.001). After 2010, complete genotype replacement was observed from non-CC92 genotypes to only CC92 genotypes. Carbapenem resistance rates were highest in AB CC92 (94.2%), followed by AB non-CC92 (12.5%) and non-*baumannii Acinetobacter* spp. (2.1%). During 2014–2017, which included clustered cases of invasive ST395, colistin resistance increased to 62.5% (n = 10/16), showing a mortality rate of 88% during this period.

**Conclusion:**

Complete genotype replacement of non-CC92 with CC92 genotypes was observed. AB CC92 was extensively drug-resistant, and pandrug resistance was observed depending on the ST, warranting careful monitoring.

## Introduction

*Acinetobacter baumannii* (AB) has emerged as one of the most problematic pathogens worldwide, causing nosocomial invasive infections in critical patients [1]. The prolonged survival of AB in hospital environments facilitates their nosocomial spread, resulting in critical infections of hospitalized patients with breaches in skin integrity or airway protection [2].

The *Acinetobacter* genus comprises four closely related species: *A. nosocomialis*, *A. pittii*, *A. calcoaceticus*, and AB, referred to as the *Acinetobacter calcoaceticus*-*baumannii* (ACB) complex [3]. Species within the ACB complex are difficult to distinguish using biochemical methods and have not been reliably identified by semi-automated commercial identification systems, such as VITEK2, which are widely used in many hospitals [4, 5]. Afterwards, the development of post-processing software for MALDI-TOF/MS identification systems led to successful identification of *Acinetobacter* spp. [6]. Accurate identification of the species within the ACB complex is required due to the dissimilar clinical significance and clinical outcome of them. A previous retrospective study demonstrated statistically significant less multidrug resistance and lower mortality of non-*baumannii Acinetobacter* spp. [7]. Thus, distinguishing AB from other species in the ACB complex is relevant for better clinical outcomes.

Carbapenems such as meropenem and imipenem are the drugs of choice for AB infections as they are active against multidrug-resistant (MDR) AB. However, carbapenem-resistant AB (CRAB) have been repeatedly reported globally for recent decades [8–10]. In the case of South Korea, the Korean Antimicrobial Resistance Monitoring System (KARMS) and the Korean Nationwide Surveillance of Antimicrobial Resistance (KONSAR) reported an alarming increase in the proportion of CRAB [11–13].

Currently, there is a paucity of clinical data, long-term observational studies, and investigations into the clinical characteristics of AB causing invasive infections in children. Therefore, the primary goal of this study was to identify the species of the ACB complex isolated from children with invasive diseases in South Korea and investigate the proportion of infections caused by AB and non-*baumannii* ACB complexes. Also, this study includes the longitudinal molecular epidemiology of AB at a single center and observes changes in the genotypes causing invasive AB infections in children over a 20-year period.

## Methods

### Study population

This study involved the prospective collection of AB isolates from patients below 19 years old diagnosed and treated for invasive AB infections at a tertiary referral children’s hospital during January 2001 to December 2020. The clinical data of the cases included were retrospectively reviewed from the electronic medical records for the following: patient demographics, underlying conditions, admission duration, cultured specimen, antimicrobial susceptibilities, treatment, and outcome of infection.

Patients that had *Acinetobacter* species isolated from sterile body fluid cultures, identified as AB (excluding ACB complex) by automated identification systems, and isolates available for storage were included as study participants. A case of invasive AB infection was defined as a patient with at least one infection sign and AB cultured from sterile body fluids (blood, pleural fluid, and ascites). In cases where multiple AB isolates were culture from sterile body fluids from one episode of invasive AB infection, only the first cultured isolate was included in the analyses.

### Isolates and DNA extraction

*Acinetobacter* spp. that were cultured from sterile body fluids of children underwent species identification and antimicrobial susceptibility testing using the following automated systems: from 2001, the MicroScan Walk-Away (Siemens Healthcare Diagnostics, Deerfield, IL, USA), from 2011, the Vitek-2 (bioMerieux, Marcy L’Etoile, France), and from August 2020, an additional M50 (BD Diagnostic Systems, Sparks, MD, USA). These isolates were collected and stored in inositol stocks at -70 °C. Collected isolates were thawed and cultured on MacConkey agar plates overnight in incubators set at 37 °C. The colonies were then collected and underwent chromosomal DNA extraction using a DNA extraction kit (DNeasy Kit; Qiagen GmbH) according to the instructions given by the manufacturer. The extracted DNA were evaluated by NanoDrop (Thermo Fisher Scientific, Inc., Waltham, MA, US) and then stored at -70 °C until use.

### Antimicrobial susceptibility testing

The results of antimicrobial susceptibility testing reported by Vitek-2 and MicroScan Walk-Away were collected. The 2016 Clinical and Laboratory Standards Institute (CLSI) guideline was used to determine the cut-off for antibiotic susceptibilities. The definition for MDR *Acinetobacter* spp. was non-susceptibility to ≥ 1 agent in ≥ 3 antimicrobial categories, and extensively drug resistant (XDR) *Acinetobacter* spp. was non-susceptibility to ≥ 1 agent in all but ≤ 2 categories, as defined by the international expert proposal for interim standard definitions for acquired resistance.

Colistin susceptibility was tested via broth microdilution (BMD) according to the CLSI-EUCAST guidelines [14]. Minimum inhibitory concentrations (MICs) were determined using untreated MicroWell trays (Thermo Fisher Scientific, Inc., Waltham, MA, US) and 5 × 10^5^ colony-forming units/mL of AB was inoculated. The plates were then incubated for 24 h and read using the Sensititre Manual Viewer (Thermo Fisher Scientific, Inc., Waltham, MA, US). According to the CLSI criteria, colistin MIC ≤ 2 µg/mL was considered susceptible, and ≥ 4 µg/mL was considered resistant [14].

### ***Acinetobacter baumannii*** genomic species identification

The discriminative partial sequence of *rpoB* gene was sequenced for genomic species identification using the extracted DNA. Partial *rpoB* gene underwent polymerase chain reaction (PCR) using the primers Ac696F (TAYCGYAAAGAYTTGAAAGAAG) and Ac1093R (CMACACCYTTGTTMCCRTGA), as reported by Gundi et al. [15]. Upon electrophoresis, the band that appeared in the 350 bp region for each of the isolates were sequenced using Ac696F and Ac1093R as sequencing primers. Using the GenBank (https://www.ncbi.nlm.nih.gov/nuccore/) website, the *rpoB* sequences of each isolate were entered and the species were identified using the BLAST search tool.

### Multilocus sequence typing of ***Acinetobacter baumannii*** isolates

All AB isolates from collected from 2001 to 2020 underwent multilocus sequence typing (MLST) by conventional sanger sequencing of the internal fragments of the 7 housekeeping genes using the oxford scheme for AB reported by Bartual et al. [16] The sequences of each loci were then submitted to the AB MLST database (https://pubmlst.org/abaumannii) and allotted allele numbers at each of the 7 loci, ultimately giving each isolate a 7-digit allele profile. This profile was then used to determine the sequence type (ST). The STs that shared 6 identical alleles of the 7 loci were clustered into a clonal complex (CC) using the eBURST (PHYLOViZ, https://phyloviz.readthedocs.io/en/latest/index.html) program.

### Statistical analyses

Categorical variables were compared by using Chi-square test or Fisher’s exact test, and continuous variables were compared by Kruskal-Wallis H test. The cox proportional hazards regression analysis was used to find the risk of mortality at 7 and 30 days after the onset of sepsis, and logistic regression analyses was used to show the inverse relation between colistin prescription and 30-day mortality. The *P* for trend was analyzed using One-Way ANOVA (analysis of variance) linear analyses and the weighted P value were considered in the analyses. All tests were two-tailed and were considered statistically significant when the P-value was < 0.05.

## Results

### Demographics and clinical characteristics

During the 20-year study period, a total of 108 non-duplicate isolates were cultured and identified as AB using commercial identification systems from sterile fluids of children admitted at Seoul National University Children’s Hospital. Of the 108 patients, 60.2% (n = 65) were male patients, and the median age was 1.4 (interquartile range, 0.1–7.9) years old. In total, 98.1% (n = 106) of the isolates were cultured from children with underlying diseases. The most common underlying diseases were non-malignant chronic diseases (n = 40, 37.0%), malignancies (n = 23, 21.3%), and congenital heart disease (n = 16, 14.8%). The isolates were cultured from the blood in 85.2% (n = 92) of the cases and from ascites fluid in 12.0% (n = 13) (Table 1).

**Table 1:** Baseline characteristics of patients with invasive *Acinetobacter* species infection

	No. of cases (%)			*P*
	Total	AB	non-AB	
	N = 108	n = 60	n = 48	
Sex, male	65 (60.2)	31 (51.7)	24 (50.0)	*0.863*
Median age (IQR), years	1.4 (0.1–7.9)	1.5 (0.1–6.8)	2.4 (0.4–11.3)	*0.164*
Underlying disease				
Malignancy	23 (21.3)	15 (25.0)	8 (16.7)	*0.293*
Congenital heart disease	16 (14.8)	11 (18.3)	5 (10.4)	*0.250*
Preterm, ELBWI	17 (15.7)	14 (23.3)	3 (6.3)	*0.015*
Solid organ transplant	10 (9.3)	5 (8.3)	5 (10.4)	*0.710*
Non-malignant chronic disease*	40 (37.0)	15 (25.0)	25 (52.1)	*0.004*
None	2 (1.9)	0	2 (4.2)	*-*
Cultured specimen				
Blood	92 (85.2)	52 (86.7)	40 (83.3)	*0.628*
Pleural fluid	3 (2.8)	3 (5.0)	0	*-*
Ascites fluid	13 (12.0)	5 (8.3)	8 (16.7)	*0.186*
Location of admission				
Ward	37 (34.3)	8 (13.3)	30 (62.5)	*< 0.001*
ICU	71 (65.7)	53 (88.3)	18 (37.5)	
Duration of admission prior to infection, median days (IQR)	14.0 (2.5–49.0)	17.0 (6.0-43.5)	9.0 (1.0–51.0)	*0.151*
Mortality				
7-day mortality	26 (24.1)	24 (40.0)	2 (4.2)	*< 0.001*
30-day mortality	32 (29.6)	28 (46.7)	4 (8.3)	*< 0.001*

AB, *Acinetobacter baumannii*, ELBWI, extremely low birth weight infant; ICU, intensive care unit; IQR, interquartile range;

* Chronic lung disease, n = 2; End stage renal disease, n = 11; Gastrointestinal disease, n = 13; Neurologic disease, n = 12; Post-CPR status, n = 2;

### Genomic species identification

All 108 isolates reported as AB via commercial identification systems underwent further species identification via partial sequencing of the *rpoB* gene. Only 55.6% (n = 60) were identified as true AB species, whereas 44.4% (n = 48) were identified as non-*baumannii Acinetobacter* spp.

**Fig. 1:**
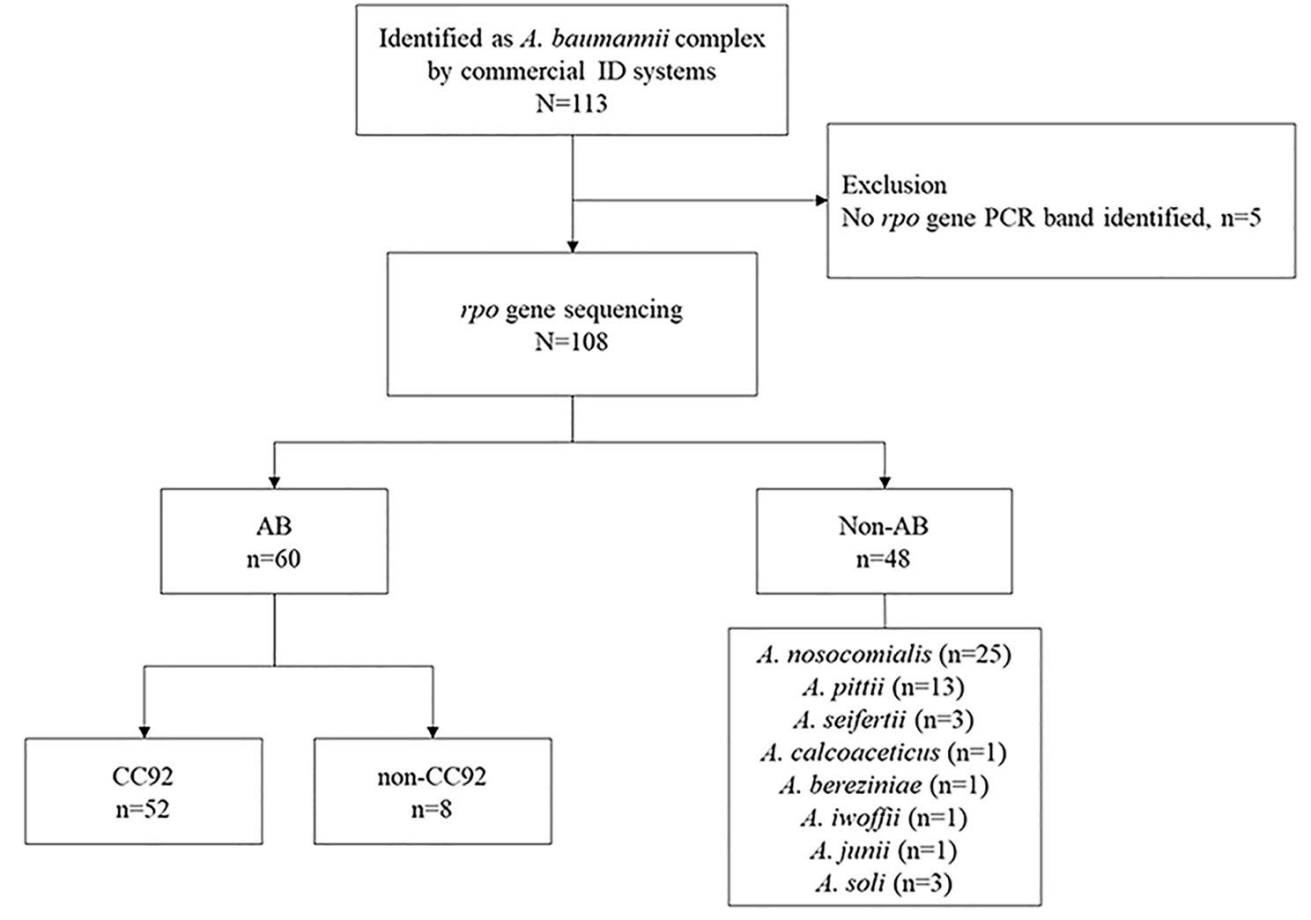
Flow chart of cases included in the longitudinal molecular epidemiology study at a single center

Of the 48 non-*baumannii Acinetobacter* spp. identified via partial *rp*o*B* sequencing, 87.5% (n = 42) of the isolates belonged to the ACB complex: *A. nosocomialis* (n = 25), *A. pittii* (n = 13), *A. seifertii* (n = 3), and *A. calcoaceticus* (n = 1). The non-ACB *Acinetobacter* spp. were identified as follows: *A. soli* (n = 3), *A. bereziniae* (n = 1), *A. iwoffii* (n = 1), *and A. junii* (n = 1) (Fig. 1).

A higher percentage of non-*baumannii Acinetobacter* spp. were isolated from patients who were in the general wards, as opposed to the intensive care unit (ICU), compared to patients with AB isolated (62.5% vs. 13.3%, respectively) (*P* < 0.001). The 7-day and 30-day mortality rates were significantly higher in patients with AB isolated (*P* < 0.001) (Table 1).

### Genotype identification of ***Acinetobacter baumannii*** isolates

**Fig. 2:**
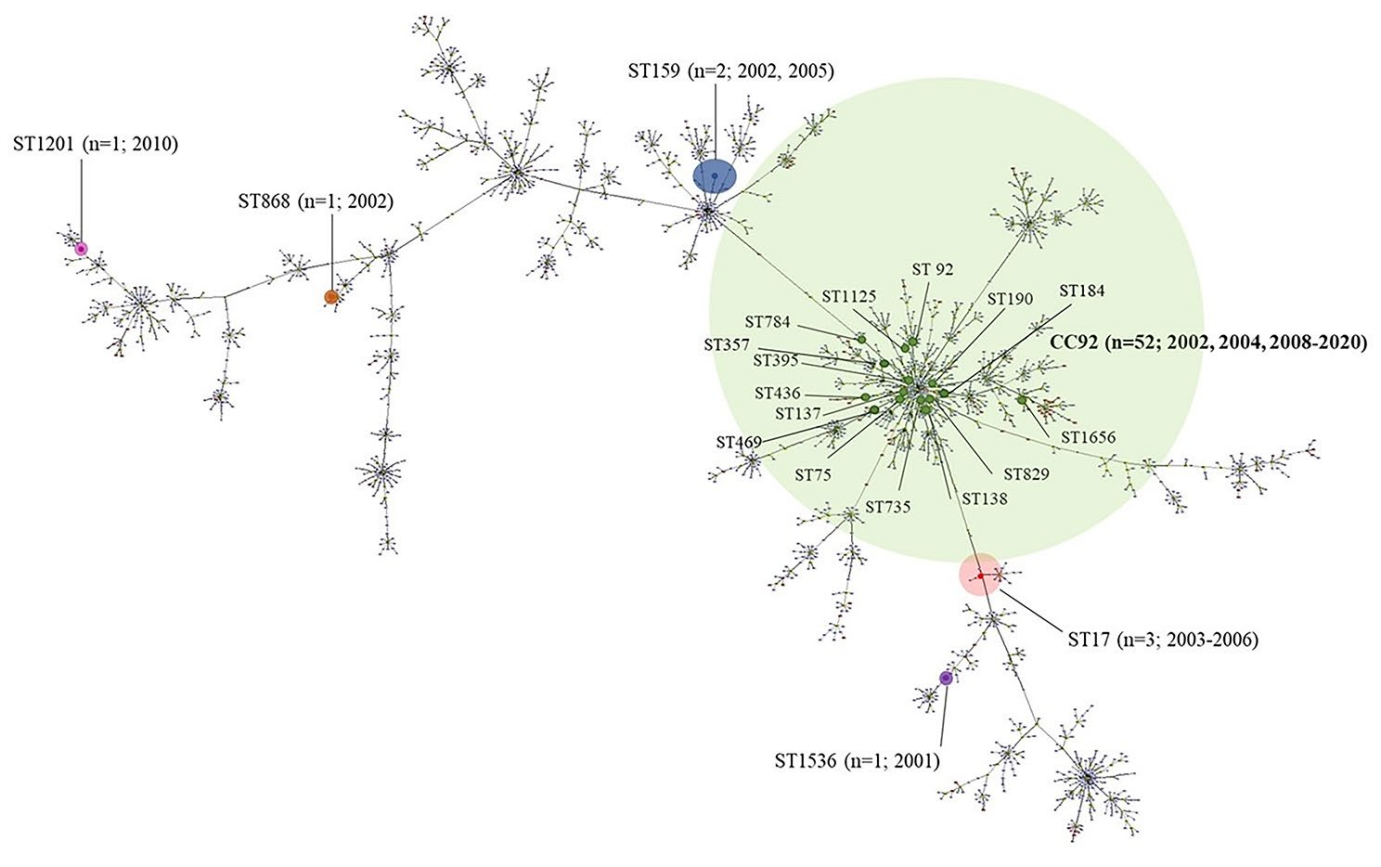
eBURST analysis of the 60 invasive AB isolates. The size of the colored shaded areas correlates with the number of isolates in the CC and STs. The nodes in the diagram represent an ST, and branches represent their relationship. The distance of the branches represents the relatedness off the STs. The number of isolates and isolated year are represented within parentheses. CC, clonal complex; ST, singleton

Isolates identified as AB species by partial *rpoB* sequencing were subjected to MLST. The phylogenetic relationships and diversification among STs are shown in the eBURST forest diagram (Fig. 2). One clonal complex, CC92 (n = 52, 86.9%), and eight singletons: ST17 (n = 3), ST159 (n = 2), ST868 (n = 1), ST1201 (n = 1), and ST1536 (n = 1) were found. Within CC92, there were 15 different STs: ST138 (n = 9), ST395 (n = 9), ST1125 (n = 4), ST75 (n = 4), ST1656 (n = 3), ST190 (n = 2), ST735 (n = 2), ST784 (n = 11), ST357 (n = 2) and one isolate each belonging to ST829, ST92, ST137, ST436, ST469, and ST184.

### Changes in species and genotype distribution during 2001–2020

**Fig. 3:**
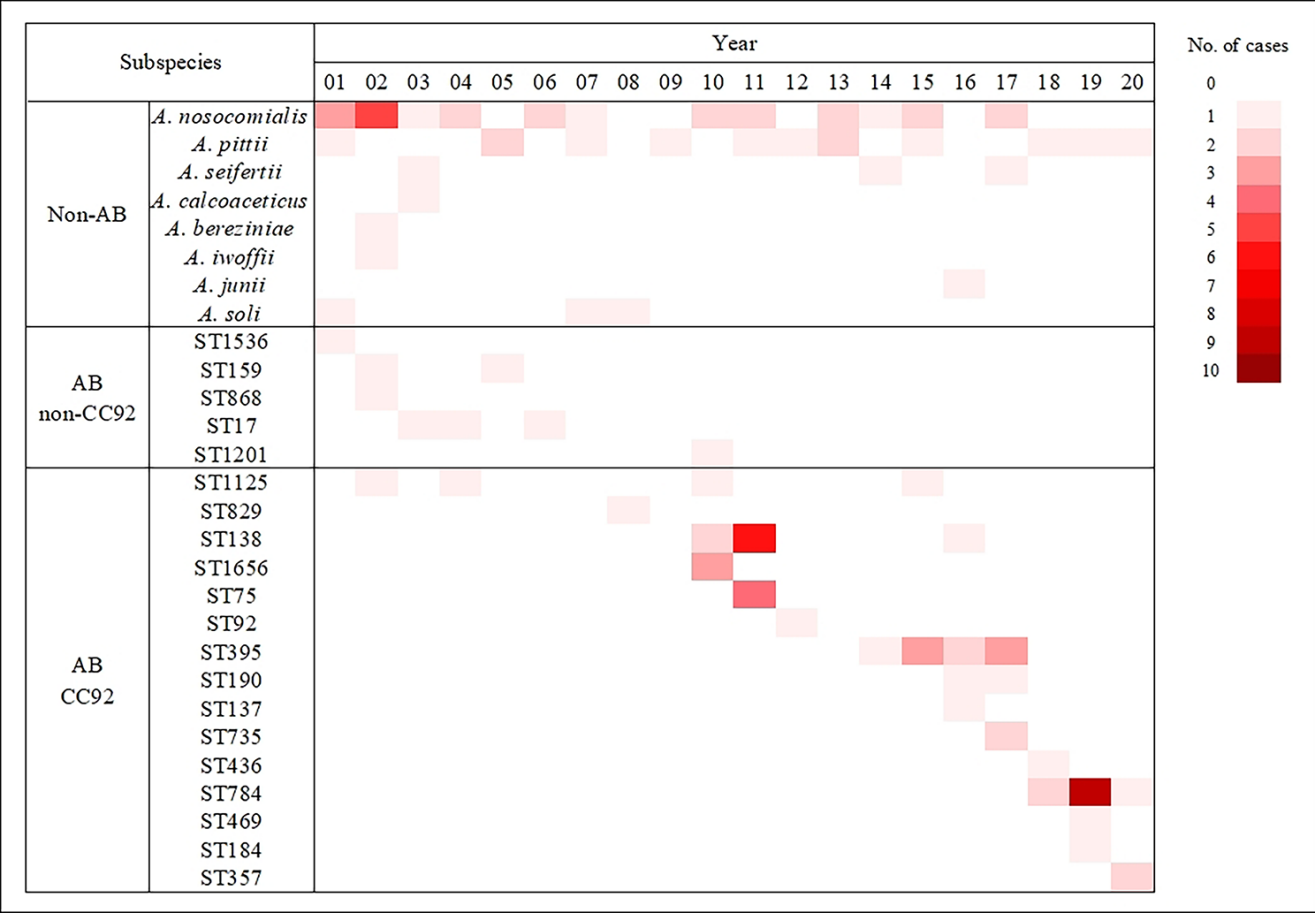
Heat map showing the number of cases of *Acinetobacter* species and genotypes isolated each year during the 20-year study period. The number of isolates are shown by the spectrum of the red color. AB, A. baumannii; CC, clonal complex; ST, sequence type;

During the 20-year study period, one cluster of cases caused by *A. nosocomialis* was observed during 2001–2002 (32.0%, n = 8/25). Subsequently, a consistent number of cases were observed throughout the study period. *A. pittii*, the second most frequent cause of non-*baumannii Acinetobacter* spp. infections, was consistently isolated throughout the study period (Fig. 3).

A distinct change in AB genotypes causing invasive infections in children was observed. During the early study period from 2001 to 2007, non-CC92 genotypes were predominant, and CC92 genotypes were intermittently isolated. However, after the 2010–2011 outbreak caused by ST138 belonging to CC92, complete genotype replacement was observed, from non-CC92 genotypes to only CC92 genotypes. The predominant strain in 2014–2017 was ST395, which changed to ST784 in 2018–2020 (Fig. 3).

### Antimicrobial susceptibility

The antimicrobial susceptibility of non-*baumannii Acinetobacter* spp. and AB non-CC92 showed higher antimicrobial susceptibility rates compared to AB CC92 (Table 2). The AB CC92 isolates showed high resistance to ceftazidime, cefepime, piperacillin/tazobactam, ciprofloxacin, imipenem, trimethoprim/sulfamethoxazole, and amikacin. Among the susceptible antibiotics, colistin showed the highest susceptibility rate of 40.4%, followed by amikacin with a susceptibility rate of 23.1%.

**Table 2:** Antimicrobial susceptibilities of *Acinetobacter* species causing invasive infections in children

	No. of cases (%)		
	Non-AB (n = 48)	AB non-CC92 (n = 8)	AB CC92 (n = 52)
Ceftazidime	41 (85.4))	8 (100)	1 (1.9)
Cefepime	43 (89.6)	7 (87.5)	1 (1.9)
PIP/TAZ	41 (85.4))	7 (87.5)	1 (1.9)
Imipenem	47 (97.9)	7 (87.5)	3 (5.8)
Amikacin	44 (91.7)	8 (100)	12 (23.1)
Ciprofloxacin	47 (97.9)	8 (100)	1 (1.9)
TMP/SMZ	38 (79.2)	7 (87.5)	6 (11.5)
Colistin	-	3 (37.5)	21 (40.4)

AB, *A. baumannii*; CC, clonal complex; PIP/TAZ, piperacillin/tazobactam;

TMP/SMZ, trimethoprim/sulfamethoxazole

Imipenem resistance rates were highest in AB CC92 (94.2%) followed by AB non-CC92 (12.5%), and non-*baumannii Acinetobacter* spp. (2.1%), showing a significantly higher resistance rate of AB CC92 to carbapenems compared to the other two groups (*P* < 0.001 for both) (Table 2).

### Changes in antimicrobial susceptibility and mortality in AB

For AB, changes in the imipenem and colistin resistance rates were investigated during the 20-year study period. Colistin-resistant AB strains were isolated as early as in 2001 (ST1536). The proportion of AB isolates resistant to imipenem was below 50.0% in 2001–2005 and at 50% in 2006–2009. However, after 2010, resistance rates increased to 94.0% in 2010–2013 and 100% in 2014–2017. Furthermore, the proportion of patients with colistin included in the treatment regimen during 2006–2009 was 0%, whereas starting 2010–2013, colistin use increased significantly (P < 0.001).

**Fig. 4:**
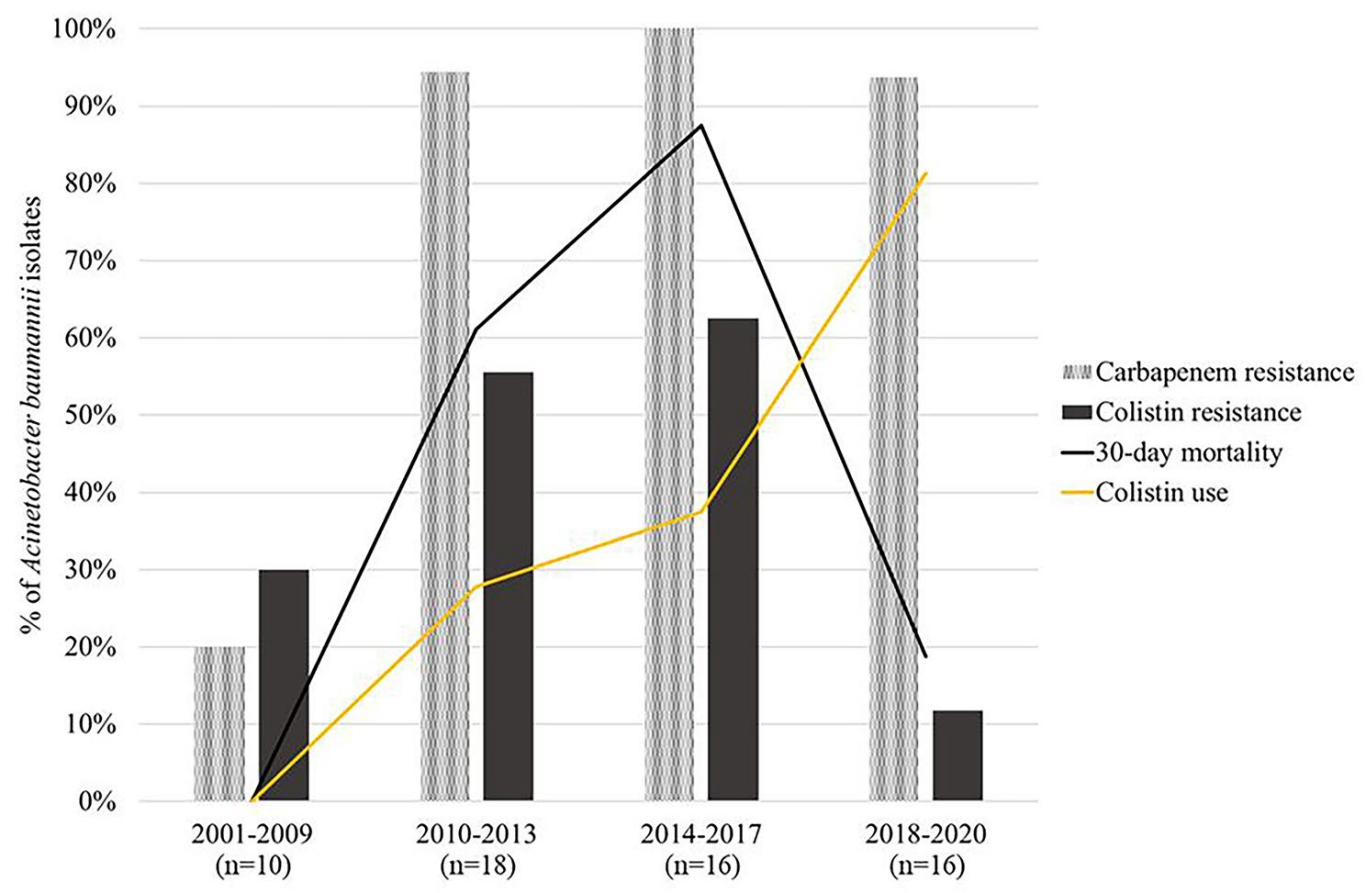
Changes in mortality rates and antibiotic resistance in *Acinetobacter baumannii* species during the study period. The study period was divided into four periods; the first period was from 2001–2009, when the dominant strains were AB non-CC92AB. From 2010 onwards, the period was divided by 4-year intervals. The P for trend is shown on the graph for each analyzed factors. AB, A. baumannii; CC, clonal complex

The colistin resistance rate was 30.0% (n = 3/10) during 2001–2005, and 0% during 2006–2009. In the period following, 2010–2013, an outbreak caused by ST138 was detected, and colistin resistance increased to 50.0% (n = 9/18). During 2014–2017, clustered cases of invasive ST395 infections were observed, and the colistin resistance rate increased further, reaching 62.5% (n = 10/16). However, during the 2018–2020 period which included an outbreak caused by ST784, colistin resistance rate decreased to 12.5% (n = 2/16). The 30-day mortality showed a similar curve, with the highest mortality observed during 2014–2017 at 88%. During 2018–2020, although the carbapenem resistance rate was similar to that in the previous time period, the 30-day mortality rate decreased to 19%. Furthermore, during the last two quinquennium, colistin prescription decreased the 30-day mortality in a statistically significant manner (OR, 15.750; 95% CI, 1.675-148.119; *P = 0.016*) (Fig. 4).

## Discussion

*Acinetobacter baumannii* has emerged as a major global threat, especially because of its exceptional ability to acquire resistance genes against all classes of antibiotics currently available. There have been high misidentification rates due to its phenotypic similarities with other *Acinetobacter* spp. However, differentiating between the species of the ACB complex, especially AB, is extremely important because of the poor outcomes and higher mortality rate in critically ill patients with invasive AB infections [17]. In our study, we found that second-generation commercial identification systems correctly identified only 55.6% of AB strains between 2001 and 2020. Before 2007, non-CC92 AB was predominant. However, after 2010, a complete replacement was observed from non-CC92 to CC92 genotypes at a single center. The AB CC92 isolates showed XDR characteristics, with only 5.8% of the isolates being susceptible to carbapenems. As carbapenem resistance increased, the proportion of patients treated with colistin also increased. The mortality rate decreased in 2018–2020 as majority of the infections were caused by colistin-susceptible ST784.

Automated phenotypic identification methods analyze reactions of the bacteria to different chemicals, creating an analytical biochemical profile, which then matches the profile to the “best fit” bacteria. First-generation automated bacterial identification systems included the analytical profile index (API) (bioMérieux, Craponne, France) and Vitek® system (bioMérieux, Craponne, France). Second-generation systems include MicroScan-Walkaway (Siemens, Munchen, Germany) and Vitek 2® (bioMérieux, Craponne, France), which are more accurate than first-generation systems [18]. However, in this study, second-generation commercial identification systems correctly identified only 55.6% (n = 47/93) of the AB isolated at a single center during 2001–2020, showing a misidentification rate of 44.4%. Similarly, a study evaluating the VITEK 2 System to identify AB showed that 68.0% of AB strains were correctly identified [19].

The correct identification of *Acinetobacter* spp. is crucial because of the poor prognosis of patients with AB infections, especially critically ill patients, compared with other non-*baumannii Acinetobacter* spp. infections [20–23]. In this study, there was a significant difference in 7-day mortality between AB and non-*baumannii Acinetobacter* spp., 40.0% vs. 4.2% (*P* < 0.001), and 30-day mortality (46.7% vs. 8.3%, *P* < 0.001). Differentiating between the species can allow clinicians to focus on early and aggressive interventions to enhance the survival and outcome of patients with invasive AB infections.

Data from Korean adults from the Korean Nationwide Surveillance of Antimicrobial Resistance (KONSAR) data in 2005 showed that the resistance rates of *Acinetobacter* spp. to imipenem and meropenem were 16% and 29%, respectively, and in 2011, the resistance rates increased to 64% and 63%, respectively [11, 12]. By 2015, the resistance rates were even higher, with the proportion of AB resistant to imipenem and meropenem being 85% and 84%, respectively [13]. Both AB and non-*baumannii* ACB complex *Acinetobacter* spp. were included in the adult data. However, in children with data including only invasive AB during similar time periods in this study, carbapenem resistance rates were similar during 2001–2009 and increased to > 90% after 2010. In the molecular epidemiology of both invasive and noninvasive ACB complexes isolated from children in Mexico, carbapenem resistance was 47% in 2017, and all XDR ACB complexes were AB, showing that carbapenem resistance is a problem in children as well as adults [24].

Pathogens identified as non-*baumannii Acinetobacter* spp. and AB non-CC92 isolates showed similar antibiotic susceptibility patterns, with susceptibility to cephalosporins, carbapenems, amikacin, and fluoroquinolones exceeding 85%, although the number of AB-non-CC92 isolates was limited. However, all AB CC92 isolates showed XDR characteristics, with complete resistance to 3rd/4th generation cephalosporins, piperacillin-tazobactam, and fluoroquinolones. Only 5.8% of isolates were susceptible to carbapenems. Susceptibility rates to colistin (40.4%) and amikacin (23.1%) were the highest, although both were below 50%.

Alarming levels of antibiotic resistance have been observed in AB worldwide. Resistance to carbapenems has been reported to reach 80.7% in Brazil, and only strains susceptible to polymyxins have been reported in Europe[25, 26]. In this study, since 2010, the increase in carbapenem resistance consequently led to increasing use of colistin in children with CRAB. However, between 2014 and 2017, a high mortality rate of up to 88% was observed in patients with invasive AB infections (Fig. 4). The high mortality can be attributed to clustered cases caused by both carbapenem and colistin-resistant pan-drug-resistant ST395. To date, there have been no previously published reports describing colistin resistance in ST395, and our study shows the need to monitor this pan-drug-resistant strain. During the consecutive period, 2018–2020, the mortality rate decreased to 19%. During this period, an outbreak of cases was caused by ST784, which was carbapenem-resistant and colistin-sensitive. However, factors associated with mortality are very complicated, as shown by the colistin resistance rate during 2001–2005, which was much higher than in 2018–2020, but with lower mortality rate. The high colistin resistance rate in 2001–2005 may have been driven by different resistance mechanisms than the colistin resistance after 2010, and further studies are needed to explain changes in mortality associated with antibiotics resistance.

The main mechanism underlying the poor outcome of children with invasive AB is their exceptional ability to acquire MDR-genes rapidly. As observed globally, there was a distinct change in the genotypes of AB causing invasive infections in children in this study. Before 2007, non-CC92 AB was predominant. However, after 2010, a complete replacement was observed from non-CC92 to CC92 genotypes at a single center. With genotype replacement, a significant increasing trend in carbapenem resistance and mortality was observed. This change has been reported globally, especially in China, where CC92 CRAB is increasing [27, 28]. Data on adults also show similar findings. A study including 19 different hospitals in South Korea reported a wide dissemination of ceftazidime resistant CC92 in South Korea [29]. During 2013–2017, wide distribution of CC92 and high prevalence of acquired carbapenemase genes among CRAB was reported in the USA [30]. Isolates collected during 2014–2015 from patients within a university hospital in southern Iran also reported the spread of closely related XDR genotypes of CC92 [31]. The epidemic dissemination of CC92 and near synchronous emergence worldwide in many countries are attributed to the successful and rapid acquisition of antimicrobial resistance genes [32, 33].

Furthermore, the pattern of circulating dominant STs within CC92 show two major patterns: in the case of ST1125, cases are observed throughout the entire study period. However, for majority of the STs, we see clustered cases or outbreaks during a certain timeframe within the study period. This latter pattern may be attributed to infection control measures applied to eradicate the ST causing clustered cases, hence elimination of the ST. However, due to the characteristics of AB, we see different STs continually emerging.

This study has several limitations. This was a single-center study; therefore, caution must be exercised in generalizing the data to other hospitals. However, our center has the largest pediatric ICU in the country, with 20 ICU beds, and is representative. Our data also show that the global trend of increase in CRAB CC92 strains applies even to hospital-acquired infections in children from a single center. Furthermore, this is the first study to investigate the 20-year long-term longitudinal molecular epidemiology of AB isolated from children with invasive infections at a single center and is therefore deemed to be valuable for monitoring and treating invasive AB infections in children. Further studies exploring the changes in resistance genes and virulence factors of these strains will provide insight into the changes in molecular epidemiology.

## Conclusions

To conclude, not only distinguishing AB from non-*baumannii Acinetobacter* spp. is important, but also identifying CC92 AB from non-CC92 AB is imperative because of their XDR characteristics. Furthermore, along with the global phenomenon, CC92 has become the dominant circulating strain in children. Many efforts are needed to prevent outbreaks in critical patients, including methods such as decreasing overall antibiotic use and relieving selective pressure, strict contact precautions, hand hygiene of medical staff to prevent transmission, and environmental disinfection to decrease sources of possible infection.

## Data Availability

The datasets used and/or analyzed during the current study are available in part from the corresponding author on reasonable request.

## Abbreviations

AB: Acinetobacter baumannii
ACB: Acinetobacter calcoaceticus-baumannii
BMD: Broth microdilution
CC: Clonal complex
CLSI: Clinical and Laboratory Standards Institute
CRAB: carbapenem-resistant AB
IQR: Interquartile range
KARMS: Korean Antimicrobial Resistance Monitoring System
KONSAR: Korean Nationwide Surveillance of Antimicrobial Resistance
MDR: multidrug resistant
MIC: Minimum inhibitory concentration
ST: Sequence type
XDR: Extensively drug resistant
